# PLR/SII/D-dimer iron triangle: a novel precision prediction strategy for *Mycoplasma pneumoniae* pneumonia-associated plastic bronchitis in children

**DOI:** 10.3389/fmed.2026.1762585

**Published:** 2026-02-25

**Authors:** Minxuan Feng, Wenyan Li, Tao Ai, Yinghong Fan, Lei Zhang, Wanmin Xia, Cheng Xie

**Affiliations:** 1 Department of Respiratory, Chengdu Women’s and Children’s Central Hospital, School of Medicine, University of Electronic Science and Technology of China, Chengdu, China; 2 Department of Respiratory, Guangzhou Women and Children’s Medical Center, Guangzhou Medical University, Guangdong Provincial Clinical Research Center for Child Health, Guangzhou, China

**Keywords:** fiberoptic bronchoscopy, lobar pneumonia, plastic bronchitis, predictive value, systemic immune-inflammation index

## Abstract

**Objective:**

Plastic bronchitis (PB) is a life-threatening pulmonary infection disease, and the early recognition and diagnostic prediction of PB are currently not well established. This study aims to identify independent risk factors for PB and develop a clinically applicable predictive model to help clinicians make earlier and more accurate judgments about the potential occurrence of PB.

**Methods:**

This study included 132 hospitalized patients with lobar pneumonia caused by *Mycoplasma pneumoniae* infection who underwent fiberoptic bronchoscopy. The study group consisted of 44 PB patients and 88 non-PB patients. Clinical data were collected and analyzed using chi-square tests, *t*-tests, non-parametric tests, Pearson *χ*^2^ tests, continuity-corrected *χ*^2^ tests, and Fisher’s exact probability tests. Univariate analysis was performed to identify potential risk factors, and logistic regression analysis was used to determine the main independent risk factors for PB. Receiver operating characteristic (ROC) curves were plotted to evaluate the predictive potential of single-factor models and a combined model of platelet-to-lymphocyte ratio (PLR), systemic immune-inflammation index (SII), and D-dimer for PB occurrence.

**Results:**

The results of univariate analysis showed that N%, L%, NLR, PLR, CRP, PCT, SII, LDH, D-dimer, and the duration of macrolide antibiotic therapy were all independent risk factors for PB. It was also suggested that continued use of macrolides after two courses did not significantly reduce the occurrence of PB. The results of multivariate regression analysis indicated that a combined analysis of PLR, SII, and D-dimer had higher predictive value for PB occurrence. This was further supported by plotting ROC curves and establishing a triad model of these indicators to achieve simple data calculation for predicting PB in clinical practice.

**Conclusion:**

This study demonstrates that PLR, SII, and D-dimer are important indicators for predicting the occurrence of PB in children. The combined model of these three indicators is more sensitive and specific than the individual risk factors in predicting PB occurrence. Additionally, this model provides synergistic guidance for the duration of macrolide antibiotic therapy.

## Highlights

- Older children have a higher risk of developing plastic bronchitis than younger ones, and this difference may be associated with a stronger immune response of older children to drug-resistant *Mycoplasma pneumoniae*.
- Chest imaging findings such as stenosis, occlusion and mucus plugs cannot effectively indicate plastic bronchitis, and imaging features are difficult to accurately diagnose this disease.
- The use of macrolide antibiotics for more than 2 courses has little effect on the treatment of plastic bronchitis (PB); when clinical symptoms or imaging changes are not significantly relieved, bronchoscopic adjuvant therapy should be adopted in a timely manner.
- Platelet-to-lymphocyte ratio (PLR), systemic immune-inflammation index (SII) and D-dimer at different elevated levels are independent risk factors for PB in children, and the higher the, multiple of D-dimer elevation, the higher the risk of PB occurrence.
- Single indicators have limitations in the diagnosis of PB, and the triple diagnostic model composed of PLR, SII and D-dimer has the optimal diagnostic value for PB.

## 1 Introduction

*Mycoplasma pneumoniae* (MP) is one of the primary pathogens of community-acquired pneumonia (CAP) in children, accounting for approximately 10–40% of pediatric CAP cases (1–9). Recent advancements in molecular diagnostic techniques and their clinical applications have deepened our understanding of the epidemiological characteristics and clinical manifestations of MP infections. While MP infections often present as mild pneumonia, some severe cases progress to lobar pneumonia with manifestations such as lung consolidation and atelectasis. Additionally, MP infections may cause various complications, including myocarditis, renal injury, and central nervous system damage (10). Among these, plastic bronchitis (PB) is a rare but potentially serious complication that has attracted increasing clinical attention (11–15). PB is defined as a pulmonary disease characterized by the formation of tree-like, gelatinous casts of varying lengths and densities within the trachea or bronchi due to various causes. It is classified as a rare acute critical illness with an unclear pathogenesis (16, 17). Depending on the extent and degree of obstruction, PB can lead to ventilation and/or gas exchange dysfunction, presenting with symptoms such as cough, wheezing, fever, chest tightness, and chest pain. Severe cases may progress to obliterative bronchiolitis, bronchiectasis, acute respiratory failure, or even life-threatening outcomes (16, 17). MP is recognized as the leading pathogen causing PB, with PB occurring even in cases where infection symptoms are mild (16). With the increasing application of bronchoscopy, reports of PB in MP-associated lobar pneumonia have grown. Some studies suggest that recurrent high fever, respiratory distress, elevated D-dimer and lactate dehydrogenase (LDH) levels, abnormal neutrophil and lymphocyte ratios, and chest imaging findings such as pleural effusion may be independent risk factors for PB (10–19). Notably, under specific epidemiological conditions such as the COVID-19 pandemic, the incidence of PB has been reported to decrease (20). Since the relaxation of COVID-19 control measures in mainland China in late 2022, a sharp increase in the prevalence of MP infections has been observed during 2023–2024. A subset of children with MP pneumonia (MPP) experienced rapid disease progression, with a significant increase in the proportion of PB cases.

This study refines the research focus to MP-infected children with lobar pneumonia (characterized by lung consolidation and atelectasis) and incorporates a retrospective analysis of macrolide and other conventional antibiotic usage, alongside novel inflammatory and immune response indices such as the neutrophil-to-lymphocyte ratio (NLR), platelet-to-lymphocyte ratio (PLR), monocyte-to-lymphocyte ratio (MLR), systemic immune-inflammation index (SII), and systemic inflammatory response index (SIRI). Furthermore, it explores their predictive value for PB. The aim is to provide new perspectives for the early diagnosis and intervention of PB in MP-associated lobar pneumonia and refine clinical indications for fiberoptic bronchoscopy to enhance diagnostic and therapeutic approaches.

## 2 Methods

### 2.1 Patients and participants

**Figure 1:**
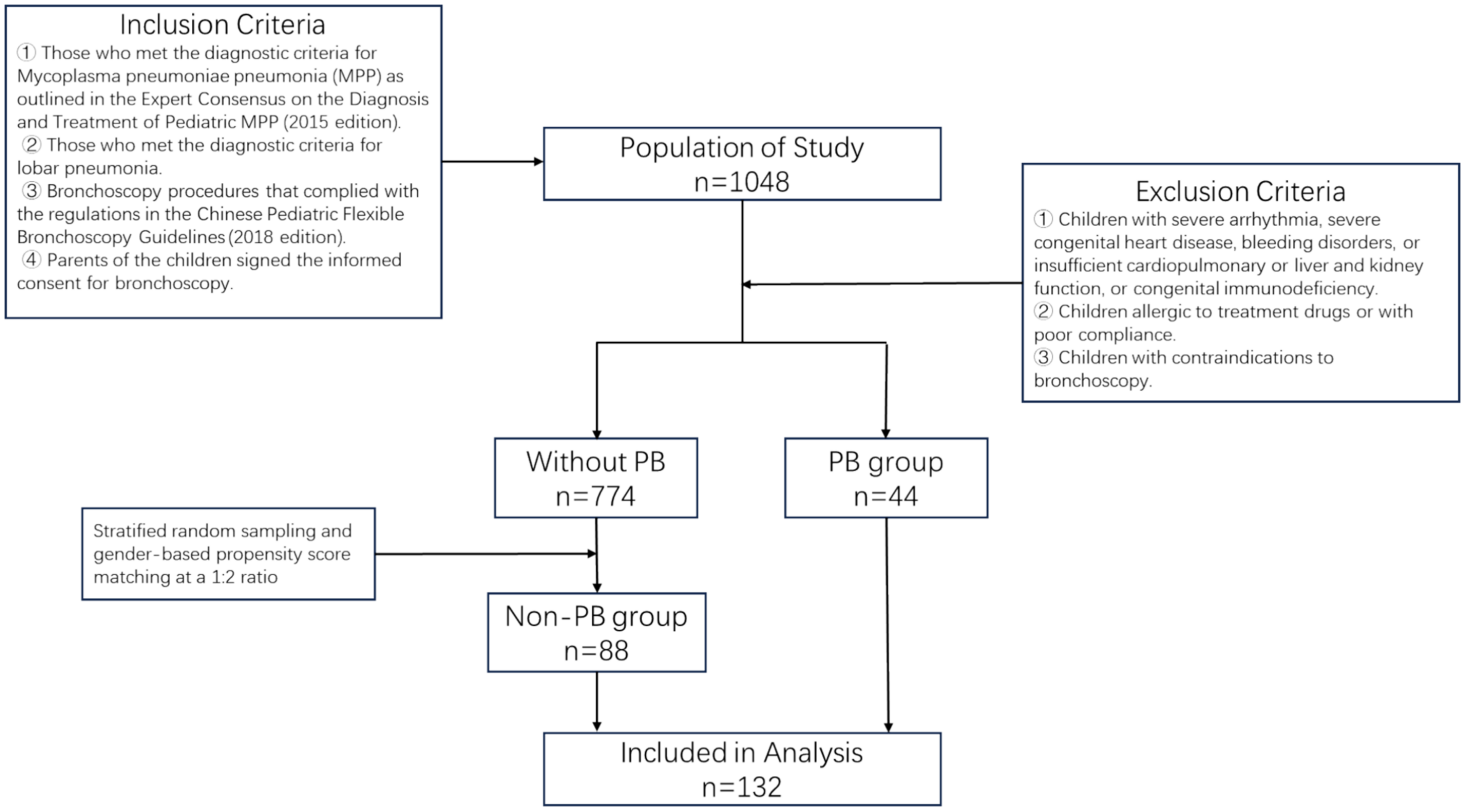
Flowchart of participant inclusion in the study.

Children diagnosed with lobar pneumonia caused by MP in the Department of Respiratory Medicine of our hospital from February 1, 2023 to January 31, 2024, who had undergone bronchoscopy were selected as the subjects of this study. The PB group consisted of all 44 children from whom plasticized materials could be removed during bronchoscopy. The non-PB group was composed of 88 children who were matched by gender structure to the PB group through 1:2 propensity score matching from over 1,000 children who met the established diagnostic criteria (i.e., “lobar pneumonia caused by MP infection and had undergone bronchoscopy”) but did not have plasticized materials. Our study has received approval from the Ethics Committee of our hospital, and all children’s guardians were required to assist in completing the informed consent form. Inclusion criteria for the children were: ① Those who met the diagnostic criteria for *Mycoplasma pneumoniae* pneumonia (MPP) as outlined in the Expert Consensus on the Diagnosis and Treatment of Pediatric MPP (2015 edition); ② Those who met the diagnostic criteria for lobar pneumonia; ③ Bronchoscopy procedures that complied with the regulations in the Chinese Pediatric Flexible Bronchoscopy Guidelines (2018 edition); ④ Parents of the children signed the informed consent for bronchoscopy. Exclusion criteria were: ① Children with severe arrhythmia, severe congenital heart disease, bleeding disorders, or insufficient cardiopulmonary or liver and kidney function, or congenital immunodeficiency; ② Children allergic to treatment drugs or with poor compliance; ③ Children with contraindications to bronchoscopy. A total of 132 children were included in the study, with 44 in the PB group and 88 in the non-PB group. The flowchart for the methods was shown in Figure 1.

### 2.2 Data collection

The indicators included in our statistical analysis are as follows: ① Duration of fever before this hospital admission; ② Duration of cough; ③ Length of preoperative illness; ④ Duration of macrolide antibiotic use before completing fiberoptic bronchoscopy; ⑤ Duration of other antibiotic use before completing fiberoptic bronchoscopy; ⑥ Pulmonary signs; ⑦ Laboratory test indicators: white blood cell count, neutrophil percentage, lymphocyte percentage, platelet count, C-reactive protein, procalcitonin, neutrophil-to-lymphocyte ratio (NLR), platelet-to-lymphocyte ratio (PLR), systemic immune inflammation index (SII), systemic inflammation response index (SIRI), lactate dehydrogenase, D-dimer, and fibrinogen content; ⑧ Co-infections with other pathogens: bacterial, viral, or mixed; ⑨ Chest imaging results.

### 2.3 Statistical method

Our statistical analysis was performed using SPSS 26.0 software. Measurement data that conform to a normal distribution are represented as *X* ± *S*, and comparisons between groups are made using the *t*-test; measurement data that do not conform to a normal distribution are represented as M (P25, P75), and comparisons between groups are made using non-parametric tests. Count data are expressed as rates, and comparisons between groups are made using the Pearson *χ*^2^ test, continuity correction *χ*^2^ test, and Fisher’s exact probability test. Multivariate logistic regression analysis is conducted for indicators with significant differences between the two groups. A *p-*value of less than 0.05 is considered statistically significant.

## 3 Results

### 3.1 The difference of demographic, general medical history, and laboratory parameters between the PB group and non-PB group

The basic demographic, general medical history, and laboratory parameters of the participants are shown in Table 1.

**Table 1:** Clinical characteristics and laboratory tests results of the 132 patients with Mycoplasma-associated lobar pneumonia.

Variables	PB group	Non-PB group	*Z*/*χ*^2^	*p*
Age	7.48 ± 2.02	6.51 ± 2.54	−2.201	0.029
Length of fever (day)	5 (4, 7)	4 (1, 6)	1.943	0.052
Length of cough (day)	6 (4, 9)	7 (5, 11)	−1.428	0.153
Preoperative course of disease			3.376	0.096
≤2 weeks	9 (20.5%)	8 (9.1%)		
>2 weeks	35 (79.5%)	80 (90.9%)		
Pulmonary signs			2.235	0.336
Normal	15 (34.1%)	42 (47.7%)		
Symmetrical with crackles	11 (25.0%)	18 (20.5%)		
Decreased breath sounds on one side	18 (40.9%)	28 (31.8%)		
Chest image				
Without stenosis, occlusion, or mucus plugs	29 (65.9%)	63 (71.6%)	0.448	0.549
With stenosis, occlusion, or mucus plugs	15 (34.1%)	25 (28.4%)		
Without pleural effusion	32 (72.72%)	56 (63.6%)	1.091	0.298
With pleural effusion	12 (27.3%)	32 (36.4%)		
Co-infection with other pathogens			5.008	0.175
None	31 (70.5%)	69 (78.4%)		
Bacteria	4 (9.1%)	5 (5.7%)		
Virus	8 (18.2%)	7 (8.0%)		
Both of the mix	1 (2.3%)	7 (8.0%)		
Preoperative use of macrolide antibiotics			4.984	0.031
<2 courses	24 (54.5%)	65 (73.9%)		
≥2 courses	20 (45.5%)	23 (26.1%)		
Preoperative use of other regular antibiotics			2.357	0.179
<1 week	32 (72.7%)	52 (59.1%)		
≥1 week	12 (27.3%)	36 (40.9%)		
White blood cell count	8.32 (5.48, 9.05)	7.59 (6.19, 9.98)	−0.936	0.349
N ratio	72.91 ± 11.94	62.06 ± 12.84	−4.685	<0.001
L ratio	15.60 (12.43, 23.58)	26.80 (20.18, 35.20)	−4.835	<0.001
Hb	122.61 ± 10.89	125.09 ± 11.10	1.216	0.226
PLT	224.00 (178.25, 299.25)	256.50 (198.25, 359.25)	−1.895	0.058
CRP	30.60 (12.47, 56.43)	14.60 (4.40, 28.03)	3.633	<0.001
PCT			23.014	<0.001
Normal	25 (56.8%)	81 (92.0%)		
Abnormal	19 (43.2%)	7 (8.0%)		
LDH	418.35 (299.30, 568.20)	267.45 (226.43, 314.38)	5.672	<0.001
D-dimer			35.541	<0.001
Normal	8 (18.2%)	59 (67.0%)		
1–10 times the maximum normal value	26 (59.1%)	28 (31.8%)		
>10 times the maximum normal value	10 (22.7%)	1 (1.1%)		
Fibrinogen			1.769	0.232
Normal	27 (61.4%)	64 (72.7%)		
Abnormal	17 (38.6%)	24 (27.3%)		
NLR	4.89 (2.68, 6.78)	2.32 (1.52, 3.47)	4.687	<0.001
PLR	200.92 (139.55, 280.02)	132.44 (93.59, 168.85)	3.91	<0.001
MLR	0.37 (0.25, 0.53)	0.29 (0.22, 0.49)	1.907	0.057
SII	1050.20 (616.07, 1893.04)	575.55 (360.67, 1034.05)	3.292	0.001
SIRI	2.12 (1.24, 3.17)	1.51 (0.84, 2.71)	1.825	0.068

*p* < 0.05 was considered statistically significant. The normal distribution are represented as *X* ± *S*, and data that do not conform to a normal distribution are represented as M (P25, P75).

PB, plastic bronchitis; Hb, hemoglobin; PLT, platelet; CRP, C-reactive protein; PCT, procalcitonin; LDH, lactate dehydrogenase; NLR, neutrophil-to-lymphocyte ratio; PLR, platelet-to-lymphocyte ratio; MLR, macrophage-to-lymphocyte ratio; SII, systemic immune inflammation index; SIRI, systemic inflammation response index.

The age difference between the two groups was statistically significant (*t* = −2.201, *p* = 0.029). The test results indicated that the children in the PB group were elder than those in the non-PB group, with an average age of 7.48 years in the PB group and 6.51 years in the non-PB group.

The duration of fever, duration of cough, preoperative course of disease, and pulmonary signs showed no significant statistical difference between the two groups of children (*p*>0.05).

There was no significant difference in the total number of white blood cells in the blood of the two groups of children. However, the ratio of neutrophils to lymphocytes in white blood cells showed a significant statistical difference. In the PB group, the proportion of neutrophils was significantly higher, while the proportion of lymphocytes was significantly lower (*p* < 0.001). The levels of CRP, PCT, NLR, PLR, SII, LDH, and D-dimer were all significantly higher in the PB group than in the non-PB group (*p*-values were <0.001, <0.001, <0.001, <0.001, 0.001, <0.001, <0.001 respectively). There was no significant difference in the comparison of other indicators (*p* > 0.05).

In addition, there was no significant difference in the proportion of patients with stenosis, occlusion, or mucus plugs in the chest imaging findings between the PB group and the non-PB group. Meanwhile, there was also no significant difference in the composition ratio of other pathogens apart from *Mycoplasma pneumoniae* between the two groups of children (*p* > 0.05).

The preoperative use of antibiotics in the two groups of children showed different results depending on the type of antibiotic. There was a significant difference in the composition ratio of children using macrolide antibiotics between the two groups (*p* = 0.031). In the category of using macrolide antibiotics for ≥2 courses, the PB group had a higher composition ratio than the non-PB group, while in the category of <2 courses, the result was the opposite. However, there was no significant difference between the two groups of children in the use of other antibiotics, such as *β*-lactam antibiotics (*p* > 0.05).

### 3.2 The independent predictive value of various quantitative indicators for PB diagnosis

**Figure 2:**
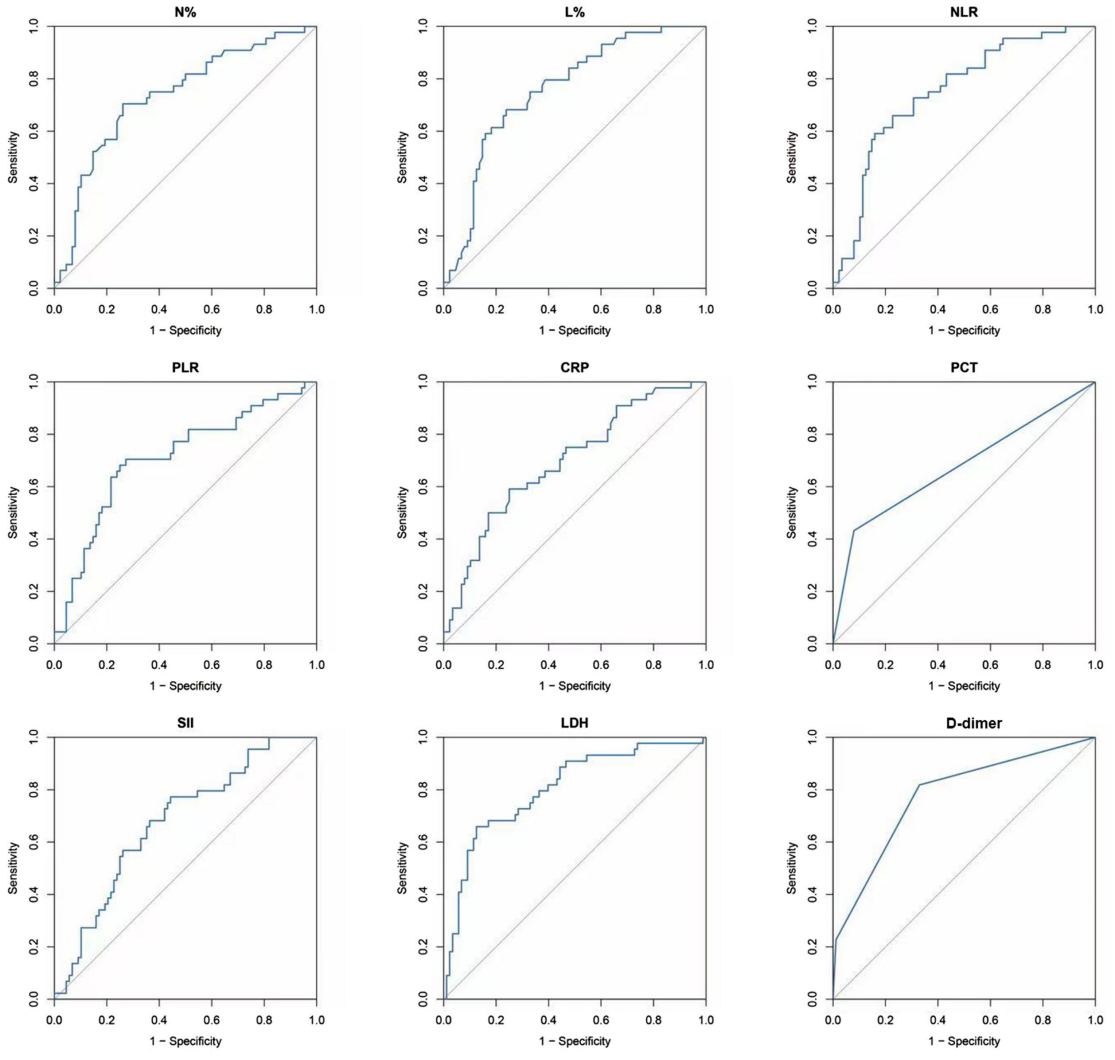
The ROC curve of N%, L%, NLR, PLR, CRP, PCT, SII, LDH, and D-dimer in predicting PB in Mycoplasma-associated lobar pneumonia. PB, plastic bronchitis; CRP, C-reactive protein; PCT, procalcitonin; LDH, lactate dehydrogenase; NLR, neutrophil-to-lymphocyte ratio; PLR, platelet-to-lymphocyte ratio; SII, systemic immune inflammation index.

From the aforementioned study, we extracted several indicators with statistical differences among blood cell-related parameters and examined their value in predicting the occurrence of PB, and the final curves were shown in Figure 2. We found that D-dimer had the highest sensitivity (81.82%, AUC = 0.7771), followed by SII (77.27%, AUC = 0.6761). PCT had the highest specificity (92.05%, AUC = 0.6761), followed by LDH (87.5%, AUC = 0.8035).

### 3.3 The multiple regression analysis of the quantifiable laboratory indicators

Subsequently, we conducted a multiple regression analysis on easily quantifiable laboratory indicators, including age, CRP, PCT, NLR, PLR, SII, LDH levels, and the ratio of D-dimer as independent variables, with concurrent PB as the dependent variable (no = 0, yes = 1), and performed a binary multiple logistic regression analysis, as they were shown in Table 2. Since some significant results may be collinear with new indicators, we adjusted for neutrophils and lymphocyte subgroups. The results showed that PLR (OR = 1.011, 95%CI = 1.002–1.020, *p* = 0.013), SII (OR = 0.999, 95%CI = 0.998–1.000, *p* = 0.044), and D-dimer (>normal value but <10 times the maximum normal value OR = 5.460, 95%CI = 2.000–14.905, *p*<0.001; >10 times the maximum normal value OR = 33.450, 95%CI = 2.879–388.676, *p* = 0.005) still had significance in the multiple analysis and were jointly related to the risk factors of PB occurrence.

**Table 2:** The multiple regression analysis of the quantifiable laboratory indicators.

Variables	*β*	Wald	OR (95%CI)	*p*
Age	−0.054	0.243	0.948 (0.766–1.173)	0.622
CRP	−0.005	0.287	0.995 (0.978–1.013)	0.592
PCT				
Normal	ref			
Abnormal	0.99	1.724	2.692 (0.614–11.803)	0.189
LDH	0.001	0.936	1.001 (0.999–1.002)	0.333
D-dimer		14.533		<0.001
Normal	ref			
1–10 times the maximum normal value	1.697	10.973	5.460 (2.000–14.905)	<0.001
>10 times the maximum normal value	3.51	7.867	33.450 (2.879–388.676)	0.005
NLR	0.226	1.799	1.254 (0.901–1.745)	0.18
PLR	0.011	6.209	1.011 (1.002–1.020)	0.013
SII	−0.001	4.053	0.999 (0.998–1.000)	0.044

*p* < 0.05 was considered statistically significant.

PB, plastic bronchitis; CRP, C-reactive protein; PCT, procalcitonin; LDH, lactate dehydrogenase; NLR, neutrophil-to-lymphocyte ratio; PLR, platelet-to-lymphocyte ratio; SII, systemic immune inflammation index.

### 3.4 The combination of PLR, SII, and D-dimer is the most valuable model on predicting PB diagnosis

**Figure 3:**
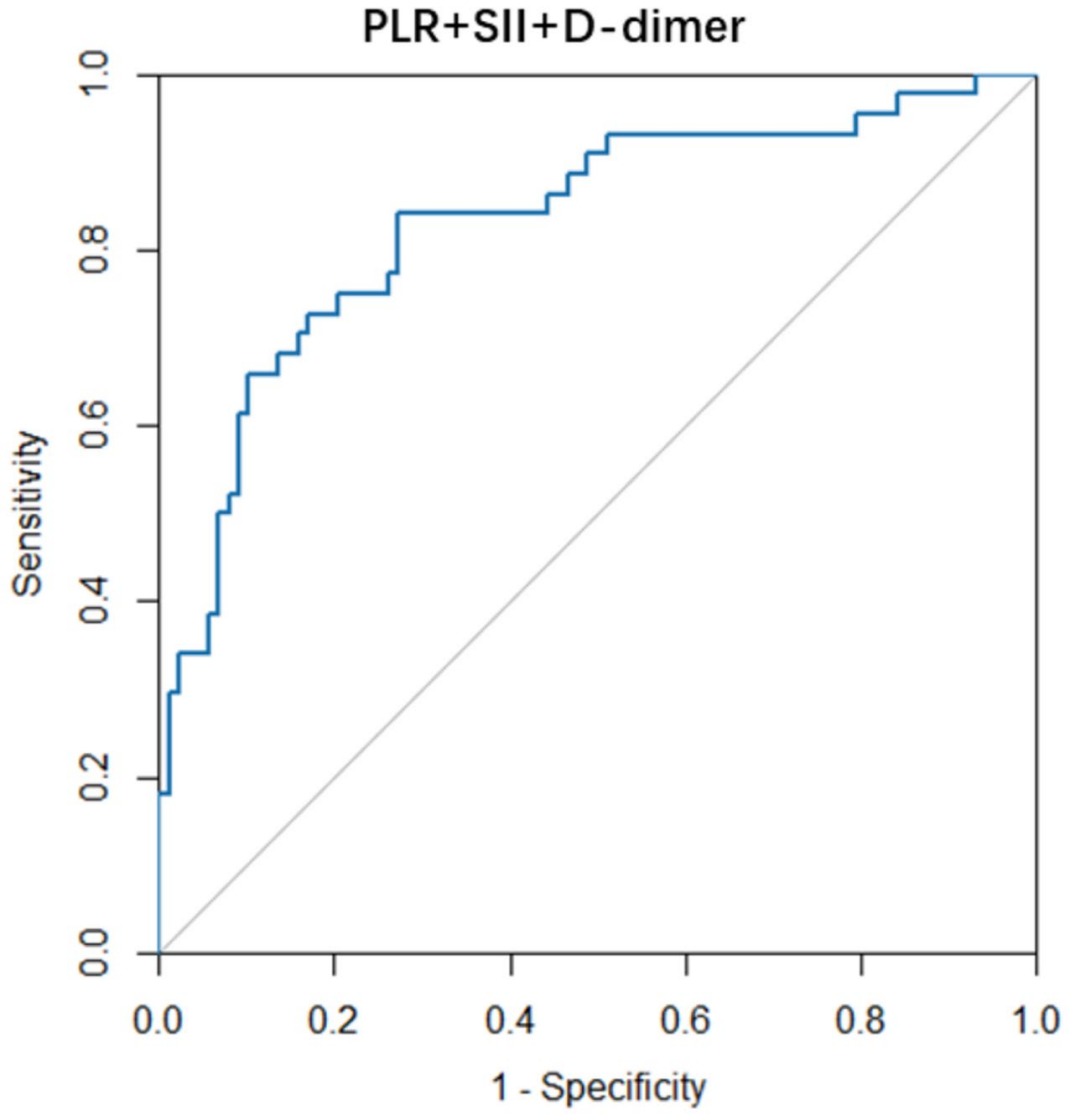
The ROC curve of the combination of PLR, SII, and D-dimer in predicting PB. PLR, platelet-to-lymphocyte ratio; SII, systemic immune inflammation index.

Since there were no indicators with both high sensitivity and high specificity in the aforementioned independent predictive indicators for PB diagnosis, we conducted another logistic regression analysis. By establishing a triad model of PLR, SII, and D-dimer for prediction, we found that this model had good diagnostic value for the formation of PB (AUC = 0.831), with a sensitivity of 84.09% and a specificity of 72.73% as shown in Figure 3. The formula for this triad model is Logit(P) = −3.251 + 0.011PLR − 0.001SII + 1.901D-dimer (> normal value but <10 times the normal value) + 4.130D-dimer (>10 times).

## 4 Discussion

The incidence of MP pneumonia (MPP) among children in mainland China has been steadily increasing, with a sharp rise in 2023–2024. Severe MPP cases often progress rapidly, initially presenting as dry, irritative cough and later as productive cough with difficulty expectorating sputum (21). Chest imaging often reveals lobar pneumonia. In severe cases, bronchoscopy may detect focal or extensive mucus hypersecretion, or secretion blockages forming casts, which can be removed as tree-like bronchial casts (21, 22).

A small-scale study from the United Kingdom suggested that PB predominantly occurs in children aged 4–12 years, with a median age of approximately 60 months (23). However, data from certain regions in mainland China indicate an onset age ranging from 7 months to 10 years (12, 24, 25). In our study, the average age of children in the PB group was 7.48 ± 2.02 years, approximately 1 year older than the non-PB group. This discrepancy may be attributed to the lack of robust bronchial wall support and difficulty in clearing secretions in younger children, coupled with stronger immune responses in older children. The slightly older average age in our study compared to previous Chinese data (12, 26) might also be linked to stronger immune responses elicited by drug-resistant MP strains (e.g., mutations in the 23S rRNA gene at positions 2063, 2064, or 2,617).

Although several studies have identified CRP, LDH, PCT, and D-dimer as markers for assessing MPP severity (19, 27–29), research on PB has also emphasized the role of chest imaging in evaluating pulmonary infection severity (20). However, in clinical practice, some PB cases lack specific chest imaging findings during the early stages, and routine, cost-effective hematological tests are often the only available diagnostic tools for outpatient children. This study seeks to identify additional, easily accessible clinical markers to aid in PB diagnosis and treatment.

In this retrospective study, we compared macrolide antibiotic usage durations. Results indicated that using macrolides for fewer than two courses was more effective in reducing PB incidence compared to prolonged usage. Continued macrolide use beyond two courses offered no significant additional benefit. Prolonged conservative treatment with antibiotics might delay disease progression in cases of persistent lung consolidation or atelectasis. Therefore, if imaging continues to show extensive consolidation or atelectasis after two courses, conservative medical management is not recommended. Comprehensive evaluations should guide the timely use of fiberoptic bronchoscopy and alveolar lavage.

Besides treatment strategies, laboratory and imaging assessments are practical methods for PB evaluation. Some studies suggest that chest imaging can assess pulmonary infection severity (11, 29–31). However, our findings indicate that imaging features such as bronchial stenosis, obstruction, and pleural effusion are insufficient for accurately diagnosing PB. This contrasts with findings by Yang et al. (20), possibly because our control group comprised children with severe pneumonia and lobar consolidation, limiting imaging’s specificity for identifying PB.

Moreover, this study uniquely compared the predictive values of NLR, PLR, SII, and SIRI for MP-PB. Among laboratory indices, a three-marker model combining PLR, SII, and D-dimer demonstrated superior predictive sensitivity and specificity compared to individual markers such as NLR, CRP, PCT, LDH, and D-dimer.

CRP and LDH are non-specific acute-phase markers of systemic infection (28, 32, 33). Elevated CRP levels, which rise within 12–48 h of inflammatory stimulation, correlate positively with MPP severity. Similarly, LDH, a marker linked to myocardial cell damage, is expressed minimally in lung tissue but may significantly increase during severe pulmonary infections. In this study, both CRP and LDH levels were significantly higher in the PB group, indicating greater pulmonary inflammatory damage. Elevated neutrophil counts in the PB group also reflect localized inflammatory responses during lung tissue damage.

Sustained inflammation can damage local blood vessels, disrupt endothelial integrity, and activate coagulation pathways, resulting in fibrinogen conversion to fibrin monomers and fibrin clots that accumulate as bronchial casts. Fibrinolysis produces D-dimer, a major fibrin degradation marker (34). Our study confirms that D-dimer is a valuable indicator of PB in children with MPP and lobar consolidation.

PCT levels reflect systemic inflammatory activity (35). In MP infections, monocytes and macrophages increase PCT secretion, leading to cellular damage and pronounced immune-inflammatory responses. Elevated PCT levels in PB cases in our study highlight heightened airway and systemic inflammatory responses.

In addition to conventional markers, indices like NLR, PLR, MLR, SII, and SIRI reflect inflammatory responses in MP infections (19, 36). For instance, NLR is a convenient marker for acute-phase inflammatory conditions, as stress responses rapidly elevate neutrophil counts while reducing lymphocyte counts (37, 38). Elevated NLR in PB cases underscores early immune-inflammatory activity, supporting its predictive value for PB.

Platelets also play crucial roles in adaptive immunity and inflammatory responses (39–41). PLR, an emerging inflammation marker, has been linked to outcomes in cancer, myocardial infarction, and sepsis (5, 42–45). Its stability makes it a valuable indicator in predicting PB, as confirmed by our findings.

SII and SIRI, novel inflammatory markers based on immune cell subtypes and platelet counts, have demonstrated associations with various diseases, including cancer, sepsis, and COVID-19 (46–49). While no prior reports have explored SII’s potential in PB prediction, our findings indicate strong sensitivity and specificity for PB.

## 5 Limitations and conclusion

### 5.1 Limitations

This study has several limitations. First, this is a single-center study. Although Chengdu is the largest and most populous city in Southwest China, its radiation scope is inevitably limited. As a city located in a basin, this study did not include participants from northern China, plateau areas, or different ethnic groups, which may lead to certain limitations in the generalizability of the study results. Second, plastic bronchitis is a relatively rare disease, resulting in a relatively small sample size in this study. In addition, due to the limited age range for eligible pulmonary function tests (e.g., forced expiratory volume in 1 s, lung diffusing capacity), inconsistent compliance of children’s caregivers, and incomplete pulmonary function data attributable to the retrospective design of this study, we did not investigate the impacts of plastic bronchitis on children’s pulmonary function, quality of life, exercise capacity, and family care-related indicators. Thus, the dimensions of outcome assessment in this study are not sufficiently comprehensive. Furthermore, conventional chest imaging examinations have no definitive diagnostic value for plastic bronchitis in *Mycoplasma pneumoniae*-associated lobar pneumonia, and the search for alternative visual imaging modalities remains warranted—this also constitutes a limitation of the present study.

### 5.2 Future directions and suggestions

Based on the limitations of this study and the clinical and research needs in the field of pediatric respirology, we put forward the following recommendations for future research. First, multicenter, large-sample prospective cohort studies should be conducted to enroll pediatric participants from different geographical regions and hospitals at all levels, expand the research coverage of plastic bronchitis (PB), and verify the generalizability of the findings of this study. Integrating artificial intelligence and machine learning technologies, a large-scale dataset should be established to further refine predictive models, which can then be formulated as expert consensus and promoted nationwide. Second, with an expanded sample size, analyses of indicators related to children’s pulmonary function, quality of life, exercise capacity and family care should be carried out, together with targeted clinical intervention studies; meanwhile, long-term follow-up should be conducted to explore whether early intervention alters clinical outcomes. A multidimensional evaluation system should be constructed to more comprehensively investigate the clinical and molecular mechanisms underlying *Mycoplasma pneumoniae* infection, lobar pneumonia and plastic bronchitis, thereby providing more reliable clinical and theoretical support for the precise diagnosis and treatment of pediatric respiratory diseases. Third, in view of the limitation that conventional chest imaging examinations have poor diagnostic performance for PB complicated with lobar pneumonia, more intuitive alternative imaging methods should be explored. For example, recent studies have shown that emerging pulmonary ventilation imaging techniques represented by hyperpolarized gas magnetic resonance imaging (MRI) (e.g., propane, butane, diethyl ether) may make up for the shortcomings of conventional chest imaging (50–53)—this technique enables direct visualization of pulmonary ventilation function with excellent spatial resolution, which may help improve the specificity of differentiating PB from other pulmonary diseases and integrate the assessment of pulmonary ventilation function with imaging findings, achieving two objectives with one measure.

### 5.3 Conclusion

In summary, neutrophil ratios, CRP, PCT, NLR, PLR, SII, LDH, and D-dimer are independent risk factors distinguishing PB from non-PB cases. Among these, the three-marker model combining PLR, SII, and D-dimer offers the strongest predictive capability for PB in children with lobar consolidation or atelectasis. This study also highlights the need for timely fiberoptic bronchoscopy when extensive consolidation or atelectasis persists beyond two macrolide treatment courses. Our findings provide robust support for clinical decision-making and enhance patient–doctor communication in managing MPP-associated PB.

## Data Availability

The datasets presented in this study can be found in online repositories. The names of the repository/repositories and accession number(s) can be found in the article/supplementary material.
